# Intestinal parasitic infections and risk factors: a cross-sectional survey of some school children in a suburb in Accra, Ghana

**DOI:** 10.1186/s13104-017-2802-7

**Published:** 2017-09-18

**Authors:** Akua Obeng Forson, Isaac Arthur, Michael Olu-Taiwo, Kathrine Korkor Glover, Prince Jonathan Pappoe-Ashong, Patrick F. Ayeh-Kumi

**Affiliations:** 1 Department of Medical Laboratory Science, School of Biomedical and Allied Health Sciences, College of Health Sciences, University of Ghana, Legon, Accra, Ghana; 2 Department of Medical Microbiology, School of Biomedical and Allied Health Sciences, College of Health Sciences, University of Ghana, Legon, Accra, Ghana

**Keywords:** Children, Parasitic infections, Risk factors, *Giardia lamblia*, Accra

## Abstract

**Objective:**

This study aimed to determine the prevalence and establish some risk factors associated with the acquisition of gastrointestinal parasitic infections in school children in Accra, Ghana.

**Results:**

The overall prevalence of intestinal parasitic infection was 15%. *Giardia lamblia* (10%) and *Schistosoma mansoni* (1.7%) were the common parasites found. Out of the 15% students postive for intestinal parasites, 13.6% had single parasites and 1.3% had double parasitic infections. Children between the ages of 4–5 and 6–7 years (20% each) had the most parasitic infections. The prevalence of intestinal parasitic infection was not significantly related to gender (p = 0.1451), and source of drinking water (p = 0.8832). However, a statistically significant association between children infected with parasites and close proximity to domestic animals or pets was observed (p = 0.0284). Continuous education on personal hygiene, environmental sanitation and deworming of domestic animals or pets are required to reduce the prevalence of intestinal parasites in school children in Accra.

## Introduction

Intestinal parasitic infections constitute a global health burden in many developing countries [1]. Parasitic diseases cause over 33% deaths globally [2]. Overcrowding, lack of clean water, and poor personal hygiene with weak nutritional status in children are known to be risk factors [3, 4].

Prevalence of parasitic infections in children has been found to vary in different countries. In Australia, some children and adults living in an Aboriginal community have been reported to have parasitic infection (89%) [5]. Whilst in China diarrhea associated parasitic infection have been reported in kindergarten and primary school children [6], in Nepal, children (4–12 years) have been reported to have parasitic infection (31.13%) [4]. Although the research from Nepal reported higher prevalence of parasitic infections in children using water from wells and rivers [4], Jacobsen et al. [7] study with young children in Ecuador have revealed that 85.7% of the children were commonly infected with either *Entamoeba histolytica* (57.1%), or *Ascaris lumbricoides* (35.5%), whilst in India a prevalence rate of 24.78% has been reported. Although previous studies in Ghana (Kumasi) have reported a 42.9% infection rate in some school children [8], limited information is available on risk factors for parasitic infections. This study aimed to fill in the gap by providing information on factors associated with the acquisition and spread of parasitic infections in school children living in low income areas in Accra Ghana.

## Main text

### Study location and design

Odododiodio constituency occupies 31.3 hectares of land along the Odaw River with a population of about 79,000 people. Some of its inhabitants are migrants from rural areas and some of them dwell in wooden structures with limited access to drinking water and sanitation [9]. A cross-sectional study was conducted with eight selected schools in the Odododiodio constituency in Accra from March, 2016 to July, 2016. Written consent was sought from parents (during Parent Teachers Association meetings) on behalf of the students. Consent forms were given to interested parents to explain the objectives and procedures. In addition, a questionnaire form was also given for socio-demographic data, environmental factors, and behavioral sanitary habits of the children.

### Participants and selection criteria

Students were only included after a signed written informed consent and filled questionnaires was obtained from the parents. Students on anti-parasitic drugs 3 weeks prior to the study were excluded. Wide neck leak proof containers were pre-labelled with name, age, sex and school and given to parents of children (2–6 years) to assist with the collection of stool samples, while children above 6 years of age were educated on the proper methods for collecting the stool samples.

### Analysis of stool samples

Stool samples were examined macroscopically for colour, consistency, presence of blood, mucus, pus and large worms [10]. The stools were examined by direct wet mount and formol-ether concentration method. For the direct wet mount, a drop of the emulsified stool samples was transferred onto both ends of a glass slide, a drop of Lugol’s iodine was added to one drop, leaving the other sample unstained. The samples were covered with a cover slip and examined first by 10× objective len, and then by 40× len of light microscope for easy and detailed identification of intestinal parasites [10, p. 108].

For the formol-ether concentrated stool, it was prepared by emulsifying approximately 1 gm of the stool with 3 ml of 10% formol-saline in a test tube [10]. The emulsified sample was seived by pouring it through layers of gauze into another test tube and then adding 4 ml of diethyl ether to the filtrate. An additional 3 ml of 10% formol-saline was added to the filtrate to reach the 10 ml mark and the mixture was mixed by inverting and shaking intermittently for 1 min. The preparation was then centrifuged at 1000 *g* for 5 mins. After, the supernatant was discarded and the sediment containing the parasites was re-suspended in 1 ml formol saline. Two slides was prepared from the concentrates and observed as described above.

### Study size

In order to achieve a 95% confidence level, the minimum study size was determined using the formula ${\text{n}} = \left( {{\text{z2}} \times {\text{p}}\left( { 1- {\text{p}}} \right)} \right)/{\text{e2}}$ where n is the sample size, z is the standard score of 95% confidence interval, p is the prevalence (since no previous data existed, 50% was used) and e is the margin of error (1.96) with significance level set at p = 0.05.

### Data handling and statistical analysis

The data were entered into Microsoft Excel and analyzed using GraphPad Prism software, version 6. In all cases, p values less than 0.05 were considered statistically significant. Initially, the association between each exposure and the presence of infection was assessed using the Chi squared test. Odds ratios were then computed to measure the strength of association.

## Results

### Socio demographic characteristics

Out of the 300 students tested, 156 (51.4%) were males and 144 (48.4%) were females (Table 1). The mean age of the study participants was 5.1 years. The minimum age recorded was 2 years and the maximum age was 9 years. The age 8–9 years recorded the highest count of students (42.7%) and age 2 years recorded the lowest count of students (7.3%).

**Table 1:** Prevalence of parasitic infections among some school children in Accra Metropolis, Ghana

	No. examined	Parasites							
		*Giardia lamblia*	*Ascaris* *lumbricoides*	*Hymenolepis* *nana*	*Entamoeba coli/dispar*	*Strongyloides* *stercoralis*	*Schistosoma mansoni*	*Taenia* sp.	Total no. (%) (n = 300)
Ages (years)									
2–3	22	4	0	0	0	0	0	0	4 (18.1%)
4–5	35	4	1	1	0	0	1	0	7 (20%)
6–7	115	15	2	1	0	0	3	2	23 (20%)
8–9	128	10	0	0	3	1	1	0	15 (11.7%)
Total no. (%)	300	30 (10)	3 (1)	1 (0.3)	3 (1)	1 (0.3)	5 (1.7)	2 (0.67)	45 (15%)
Sexes									
Male	156	17	2	1	3	0	3	2	28 (17.9%)
Female	144	13	1	0	0	1	2	0	17 (11.8%)

### Prevalence of intestinal parasites

Of the 300 stool samples examined, 45 (15%) students were positive for one or more intestinal parasites. Among these, a total of 28 males and 17 females were (Table 1). Among the different ages, the least to the most infected students with the parasites were 8–9 years (11.7%), 2–3 years (18.1%), 6–7 years (20%), and 4–5 years (20%). The prevalence of *Giardia lamblia* infection was 10%, followed by *Schistosoma mansoni* (1.7%), *A. lumbricoides* (1%), *E. histolytica*/*dispar* (1%), *Hymenolepis nana* (0.3%) and *Strongyloides stercoralis* (0.3%) (Table 1). Out of 45 students positive for intestinal parasites, 41 (13.6%) had single and 4 (1.3%) had double parasitic infection in three males and one female.

### Intestinal parasites and possible risk factors

One hundred and twenty-two children were found to be living in close proximity to some pets or domestic animals (Table 2). The odds of intestinal parasitic infection in children who live in close proximity to animals is 0.47 higher than those who do not (p = 0.0284). The prevalence of intestinal parasitic infections revealed a statistically significant association for children who lived in close proximity to domestic animals or pets.

**Table 2:** Univariate analysis of intestinal parasitic infections and potential risk factors among children in schools in Accra Metropolis Ghana

Risk factors	Positive (n = 45)	Negative (n = 255)	Total N = 300 (%)	p value	OR (95% CI)
Gender					
Male	28	126	154 (51.3)	0.1451	1.686 (0.8795–3.233)
Female	17	129	146 (48.1)		
Domestic animals or pet in the house					
Yes	27	194	221 (73.7)	0.0284*	0.4716 (0.2432–0.9147)
No	18	61	79 (26.3)		
Source of drinking water					
Pipe borne water	38	213	251 (83.7)	0.8832	
Packed sachet water	5	26	31 (10.3)		
Rain water	2	16	18 (6)		
Toilet facilities					
Latrine	37	228	265 (88.3)	0.1163	
Water closet	1	10	11 (3.7)		
In water bodies, bush or refuse dump	7	17	24 (8)		
Close proximity to refuse dump					
Yes	38	223	261 (87)	0.6301	0.7790 (0.3207–1.892)
No	7	32	39 (13)		
Shoe wearing habit					
Yes	43	235	278 (92.7)	0.4201	1.83 (0.4125–8.118)
No	2	20	22 (7.3)		
Hand washing habits					
Yes	43	239	282 (94)	1	1.439 (0.3193–6.488)
No	2	16	18 (6)		
Source of food					
Cook in the house	21	100	121 (40.3)	0.4103	1.356 (0.7169–2.566)
Street food	24	155	179 (60)		

Note * = p < 0.05

Two hundred and eighty-two (94%) of the children reported they washed their hands with soap and water before meals and after defecation. The odds of intestinal parasitic infection in children who do not practice hand washing before eating was 1.43 times higher than those who practice it (p = 1.0, 95% CI 0.3193–6.488) (Table 2).

Further analyses of the data revealed that intestinal parasitic infection was not dependent on the type of used toilet facilities (p = 0.1163) and source of food (p = 0.4103) or drinking water (p = 0.8832). Furthermore, no statistically significant associations were observed between male versus female (p = 0.1451). Although there was no statistically significant association between age and prevalence of parasitic infection, the age group 2–3 years had a slightly high prevalence (18.1%) of parasitic infection compared to age groups 4–5 and 6–7 years.

## Discussion

This study reports for the first time risk factors associated with the acquisition of parasitic infections in school children living in over crowed suburbs in Accra. The study revealed an overall prevalence of 15.1% for intestinal parasites, which is lower than the 72.9–83.8% reported in Ethiopia [11, 12]. The differences in results may be due to varying environmental conditions or ongoing yearly deworming program of under-fives employed by the Ghana Ministry of Health in school children [13].

Compared to the parasitic infection in females (11.8%), males were found to have a slightly high prevalence (17.9%). This prevalence is lower than the 32.1 and 35.9% for females and males reported in Ethiopia [14]. The differences in prevalences may be because males have access to play in parks than their females who are often engaged in the household chores [15]. In addition, a few students within the 2–3 year group participated in this study, but 18% were found to be infected with *G. lamblia* (Table 1). The total prevalence for *G. lamblia* (10%) in this study is lower than the 53% reported in Mexico [16], but it’s higher than the 6.5% reported in Morzanbique [17].

Legesse and Erko’s [18] study in Ethiopia have reported mixed infection with two parasites (23.2%) and three parasites (5.3%) in some school children. Whilst Abera and Nibret [19] have reported single (35.1%), double (8.4%) and triple infection (0.8%) in children in Ethiopia. This study found only four students (three males and one female) with mixed parasitic infections. The varying differences in mixed infections may be due differences in concentrations of parasites and sanitation conditions of the communities [20].

In Saudi Arabia, Majed [21] reported 8.4% prevalence for *S. mansoni*. Whilst Gelaw et al., [14] reported 34% for *S. mansoni* in school children in Ethiopia, this study found a low (1.7%) prevalence for *S. mansoni*. Furthermore, Abera et al., [19] have reported 5.9% for *A. lumbricoides* in some children in Ethiopia, whiles Kamga et al., [22] in Cameroon reported 24.9%. These findings are higher than the results obtained in this study (1%). In Nepal, Bishnu et al., [4] reported 46.56% and In Pakistan, Wadood et al., [23] reported 34% for *H. nana* in children. These findings are higher than the 0.3% observed in this study. Although in conformity to Gelaw et al., [14], 0.3% prevalence was reported for *S. stercoralis*, the low prevalences of helminths infections in this study may be due to differences in shoe wearing habits of the children and ongoing yearly deworming program by the Ministry of Health of Ghana [9].

Although it was observed that sanitation and water facilities in the schools were inadequate, statistically, parasitic infection was independent of gender, hand washing or shoe wearing habits and source of food eaten by the children. The absence of drinking water in schools may drive pupils to other unhygienic sources (e.g. wells) thereby increasing risks of parasitic infections [23–26]. However, children who live in close proximity to domestic animals or pets were more susceptible to infection (p = 0.0284) in this study. This is in contrast to Gelaw et al. [14] study which reported hand washing after defecation was associated with the acquisition of intestinal parasites. De-worming of house hold pets is therefore recommended to help reduce the prevalence of parasitic infections.

## Conclusion

The acquisitions of intestinal parasites in school children were dependent on living in close proximity to domestic animals. Therefore, there is the need for continuous mass deworming programs (for pets and children) and hygiene education of teachers, students and parents is required.

## Limitations

The present study was subjected to the following limitations; as the collection period was short, potential seasonal fluctuations were not evaluated to determine potential impact of parasitic infections at different seasons. The study is the first of its kind with school children in Odododiodio constituency in Greater Accra and we were restricted by Ghana Education Service to this small population size. A bigger survey that will incorporate treatment and other variables is planned pending appropriate funding. Other includes lack of baseline data on if the students have ever been infected or treated for parasitic infections.
